# Transcriptome sequencing, annotation and polymorphism detection in the hop bush, *Dodonaea viscosa*

**DOI:** 10.1186/s12864-015-1987-1

**Published:** 2015-10-16

**Authors:** Matthew J. Christmas, Ed Biffin, Andrew J. Lowe

**Affiliations:** Environment Institute and School of Biological Sciences, The University of Adelaide, North Terrace, Adelaide, 5005 SA Australia

**Keywords:** *Dodonaea viscosa*, *de novo* assembly, RNA-seq, SNP, Gene ontology

## Abstract

**Background:**

The hop bush, *Dodonaea viscosa*, is a trans-oceanic species distributed oversix continents. It evolved in Australia where it is found over a wide range of habitat types and is an ecologically important species. Limited genomic resources are currently available for this species, thus our understanding of its evolutionary history and ecological adaptation is restricted. Here, we present a comprehensive transcriptome dataset for future genomic studies into this species.

**Methods:**

We performed Illumina sequencing of cDNA prepared from leaf tissue collected from seven populations of *D. viscosa* ssp. *angustissima* and *spatulata* distributed along an environmental gradient in South Australia. Sequenced reads were assembled to provide a transcriptome resource. Contiguous sequences (contigs) were annotated using BLAST searches against the NCBI non-redundant database and gene ontology definitions were assigned. Single nucleotide polymorphisms were detected for the establishment of a genetic marker set. A comparison between the two subspecies was also carried out.

**Results:**

Illumina sequencing returned 268,672,818 sequence reads, which were *de novo*assembled into 105,125 contigs. Contigs with significant BLAST alignments (E value < 1e^-5^)numbered at 44,191, with 38,311 of these having their most significant hits to sequences from land plant species. Gene Ontology terms were assigned to 28,440 contigs and KEGG analysis identified 146 pathways that the gene products from 5,070 contigs are potentially involved in. The subspecies comparison identified 8,494 fixed SNP differences across 3,979 contiguous sequences, indicating a level of genetic differentiation between them. Across all samples, 248,235 SNPs were detected.

**Conclusions:**

We have established a significant genomic data resource for *D. viscosa*,providing a comprehensive transcriptomic reference. Genetic differences among morphologically distinct subspecies were found. A wide range of putative gene regions were identified along with a large set of variable SNP markers, providing a basis for studies into the evolution and ecological adaptation of *D. viscosa.*

**Electronic supplementary material:**

The online version of this article (doi:10.1186/s12864-015-1987-1) contains supplementary material, which is available to authorized users.

## Background

*Dodonaea viscosa* (L.) Jacq. (hop bush) is a dioecious woody shrub with a worldwide distribution across six continents, spanning from 33°N (in California) to 44°S (in New Zealand’s South Island). The species evolved on mainland Australia [1] and its wind-dispersed seeds are thought to be capable of traversing long distances over oceans due to their high physical dormancy [2]. In a flotation experiment, West showed that 30 % of seeds were still afloat after 100 days, and high germination rates were still found after immersion in seawater for 6 months [3]. Hop bush is very widely distributed across a broad range of ecosystems and exhibits high levels of phenotypic variation. As a result, *D. viscosa* has been split into seven subspecies, as characterised by JG West [4], based mainly on leaf characteristics and capsule morphology. Three of the subspecies, *viscosa, burmanniana,* and *angustifolia,* have extra-Australian distributions, whereas ssp. *angustissima, cuneata, mucronata,* and *spatulata* are all found only within Australia.

In Australia, *D. viscosa* is a common species found in a wide range of habitat types, from temperate woodlands to desert gullies and arid shrublands. It has high ecological significance and is widely used in revegetation projects. The high level of phenotypic variation in this species along with its ecological amplitude makes it an ideal species for investigating species divergence and adaptation to local conditions. Within South Australia, *D. viscosa* spans a steep rainfall and temperature gradient within the Adelaide Geosyncline, with ssp. *angustissma* and ssp. *spatulata* restricted mostly to the north and south of this region respectively. Average annual rainfall varies from 200 mm at the northern extreme of the Flinders Ranges to 700 mm on Kangaroo Island in the south, and mean maximum temperature of the warmest month ranges from 23 °C in the south to 36 °C in the north. Within this region it has been demonstrated that *D. viscosa* ssp. *angustissima* exhibits a cline in leaf width, with narrower leaves at lower latitudes [5, 6]. Through the use of herbarium specimens dating back 127 years, GR Guerin, H Wen and AJ Lowe [6] also showed that average leaf width had decreased over that time period. Narrower leaves are an adaptation to hotter, drier climates with broader leaves being more susceptible to extremes in temperature [7]. As a result, GR Guerin, H Wen and AJ Lowe [6] suggest that this shift in leaf width over space is an adaptation to climate, and temporal shifts in leaf width are a response to historical climate variation. However, with only morphological data from field and herbarium specimens used to demonstrate this correlation, whether the observed patterns are a result of a plastic or a genetic adaptation response to climate is unknown. The genomic data presented here will act as a starting point to addressing these questions.

Despite the rapid increase in sequencing capabilities over the last decade, there is currently a lack of genomic data available for this genus. A search on the NCBI nucleotide database with the search term “*Dodonaea*” returned 182 results for the genus, with 146 for *D. viscosa* specifically (March, 2015). The vast majority of these data are barcode markers (e.g. nuclear, ribosomal ITS, plastid matK and rbcL) for phylogenetic studies. In order to identify and investigate potential genes underlying the phenotypic clines demonstrated in this species, genomic resources must first be developed.

For non-model species, RNA sequencing (RNA-seq) is now regularly utilised as an effective method for generating a reduced representation of a species’ genome, specifically targeting the transcribed portion of the genome [8–17]. An advantage of transcriptome data, particularly when looking to address questions of adaptation, is that the majority of the transcriptome sequences generated will be from coding regions and therefore of potential functional importance. A major hurdle with the use of this type of data for non-model organisms is that, in the absence of a reference genome, transcript sequences must be assembled *de novo*. With billions of short reads to work with this is no mean feat, and requires a large amount of computational power along with robust, reliable algorithms. As a result, a suite of *de novo* assemblers have now been developed for this purpose [18]. However, depending on the assembler used, results can vary in terms of number and length of contigs [11, 19]. In comparison to assembly against a reference genome, *de novo* assemblies require higher coverage in order to accurately assemble contigs and, as there is no reference, sequencing errors and the presence of chimeric molecules can have a much greater impact [20, 21]. Bearing these issues in mind, being stringent on base quality thresholds and only using contigs with highly significant BLAST hits (an e-value of ≤1e^−4^ is commonly used throughout the literature [22]) to previously published, putatively homologous sequences (e.g. those found in NCBI non-redundant and Swiss-Prot databases) will help to ensure high confidence in the resultant assembly. In addition, an approach that makes use of more than one assembler and then compares the resultant assemblies by looking for shared and therefore potentially more robust contig calls derives an assembly of high confidence in the absence of a reference genome [23].

In this study, we characterise the leaf transcriptome of *Dodonaea viscosa* with the aim to identify and functionally annotate a large number of expressed genes as well as identify single nucleotide polymorphisms between populations collected along a latitudinal gradient for development as molecular markers. The outcomes from this study will be used to set the stage for future studies into the population and adaptation genomics of this species, with the developed marker set being utilised to further our understanding of adaptive variation along an environmental gradient. Our study presents a valuable resource for on-going research into this ecologically variable and significant species.

## Results and discussion

### Sequencing and assembly

Illumina Hiseq sequencing of seven cDNA libraries prepared from leaf mRNA generated 268,672,818 sequencing reads, totalling 26.86 Gb. 147,494,172 reads were from ssp. *angustissima* and 121,178,646 were from ssp. *spatulata***.** Following quality control steps of removal of duplicates and trimming of sequences on length and quality, a total of 227,376,588 reads remained. 72.3 % of the reads were assembled using CLC Genomics Workbench v. 6 (CLC; http://www.clcbio.com/products/clc-genomics-workbench/) into 105,125 unique contiguous sequences (contigs) of mean length 615 bases (N50 = 812) with a total of 65,390,455 bp. The smallest and largest contigs were 201 and 15,009 nucleotides respectively. 133,969,990 of the reads included in the assembly remained in pairs, whereas 27,204,111 were broken pairs. All reads were then mapped back to the assembly, with 175,102,401 successfully mapping and 52,274,187 not mapping.

Of the 227,376,588 cleaned reads, only 72.3 % were incorporated into contigs in the *de novo* assembly. The remaining 27.7 % were therefore not included in any further analysis as, at only 100 bp long, they were too short to be considered on their own. There are a number of possible reasons as to why these sequences were not incorporated into contigs. For example, the presence of short microRNA, degradation of RNA during the extraction process, sequencing errors, low sequence coverage, contamination from other organisms, the assembly algorithm used (see below), and low level expression of certain transcripts could all lead to sequences being omitted during the assembly [18, 24].

An expanding number of programs exist for the *de novo* assembly of short-read transcriptome data [18]. Attempting to assemble a transcriptome from short sequences without a reference genome is not a simple task computationally and, as a result, none of these assemblers claim to be perfect. Some of the issues faced are that high levels of coverage are required (over 30×) thus excluding transcripts with low expression [21] and the assembly process is very sensitive to sequencing errors and chimeric molecules [20]. Comparisons of assembler performance have shown that assemblies will vary depending on the assembler used in terms of number of contigs generated, length of contigs, and resultant BLAST success [11, 19]. We elected to use the CLC *de novo* assembler as it has performed well in a number of previous Illumina-based transcriptome assemblies [25, 26] and is the assembler of choice in [22].

In an attempt to validate the contigs produced using CLC the reads were also assembled through the Trinity pipeline [27]. This generated 208,604 contigs. Following removal of duplicates 185,384 contigs remained. Trinity is very effective at identifying splice variants, however this results in a high proportion of redundancy in the data compared to the CLC assembly [23]. It has been demonstrated that CLC is one of the least redundant assemblers [19]. In order to reduce this redundancy in the Trinity assembly the longest contig per component or putative gene was selected. This gave a total of 94,758 contigs, which is comparable in number to the CLC total of 105,125. Reciprocal mappings of the two sets of contigs showed there to be 79.25 % overlap between them. This demonstrates that the two independent assembly algorithms can give highly similar outputs, thus providing more confidence in the resultant set of contigs. All CLC contigs were used in the downstream analysis, rather than just those in common with the Trinity assembly, so as not to lose any potentially useful information.

### Sequence annotation

Of the 105,125 CLC-generated contigs, 44,191 (42 %) had significant alignments (≤1e^−5^) to sequences in the NCBI’s non-redundant database. As has been the case in previous transcriptome assemblies, contig length was a significant predictor of the presence or absence of a significant BLAST hit (logistic regression, slope = 0.104, intercept = −4.037, P < 2e^−16^) [14, 26]. Mean length of contigs with significant BLAST hits was 888 bp (max = 15,009, min = 201, SD = 877) and for those without significant BLAST hits was 421 bp (max = 5330, min = 201, SD = 262). 58 % of the contigs had no homologous sequences within the nr database. This could be for a number of reasons, such as the presence of untranslated mRNA, chimeric sequences resulting from assembly errors, sequences from uncharacterised genes, and sequences from genes unique to *D. viscosa*.

**Fig. 1:**
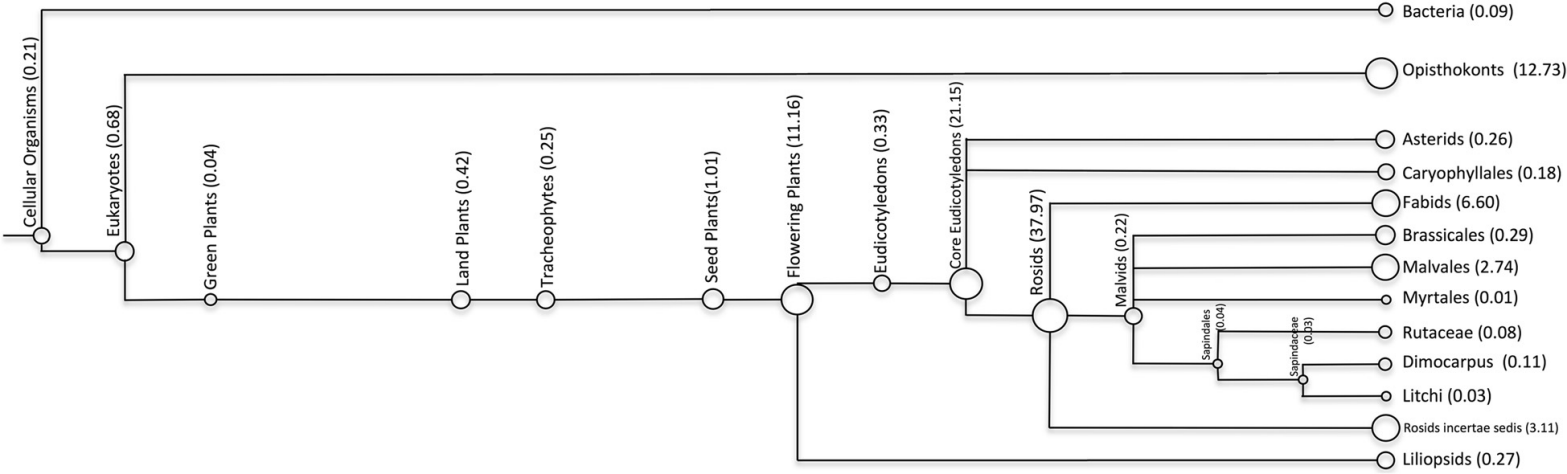
Most recent common ancestors of assembled contigs. Output from MEGAN, where the output from BLASTX searches of assembled contigs against the NCBI nr database was used to assign contigs to a taxonomic node. The size of each circle reflects the number of contigs assigned to each node, with the percentage of assigned contigs indicated in parentheses

Thirty-eight thousand three hundred eleven (86.7 %) of contigs with significant BLAST hits had their top hit to a species within the Viridiplantae (Fig. 1). The remaining 13.3 % contigs had top BLAST hits to fungi (10.3 %), arthropoda (2.8 %), bacteria (0.09 %), or viruses (0.01 %). As these were field-collected samples with high risk of contamination from endophytes, parasites, and symbionts it was not surprising that a proportion of contig sequences had significant BLAST hits to non-plant sequences (13.3 % of contigs with significant BLAST hits). Taking the contigs with significant blast hits to fungal species, it was interesting to note that the fungal species represented in the data were very similar across all samples. *Ceriporiopsis subvermispora* and the sac fungus *Baudoinia compniacensis* were the most prevalent in all samples, with 13.7 and 13.9 % of the contigs identified as fungal blasting to homologous sequences with these species respectively. The microbial communities associated with *Dodonaea viscosa* are largely unknown and these data could provide a starting point for future research into this.

**Fig. 2:**
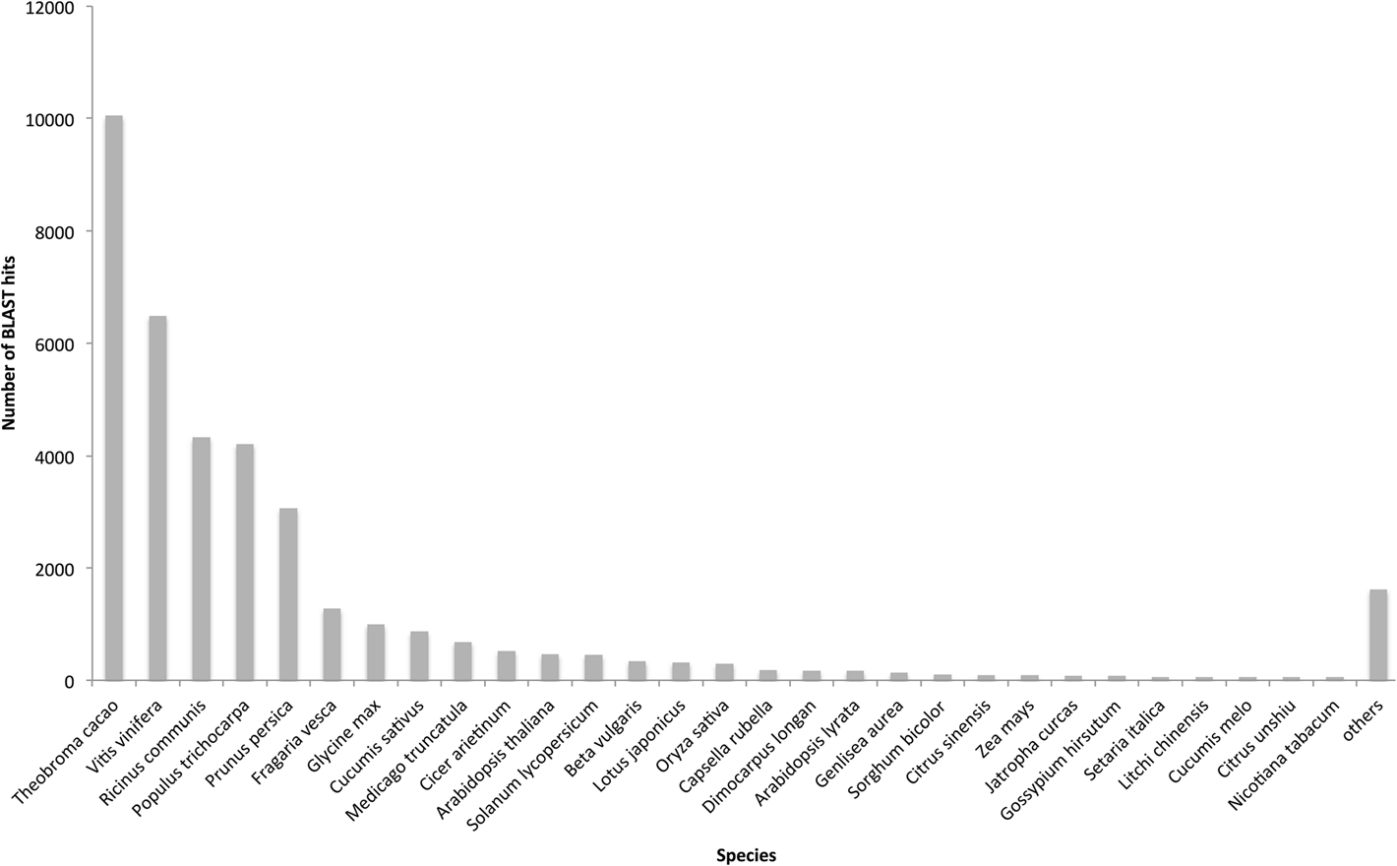
Top BLAST hit species. Species from the NCBI nr database with the top BLAST hits (lowest e-value) to the assembled contigs. Species are listed on the x-axis in rank order according to their total number of top BLAST hits (y-axis)

All contigs without a significant blast hit to plant sequences were excluded from any further analysis, leaving 38,311 contigs remaining. Within these 38,311 contigs, 30,027 unique protein accessions were identified from the BLAST results. The most closely related species to *D. viscosa* with sequenced genomes are *Citrus clementina* and *Citrus sinensis*, also members of Sapindales. The *Citrus sinensis* genome contains an estimated 29,445 protein-coding genes [28], suggesting that our transcriptome is a good representation of the genes present within *D.viscosa*. The most frequent BLAST hit species was *Vitis vinifera* with 108,515 hits. *Theobroma cacao* had the second most frequent hits, with 60,718*.* In terms of the top BLAST hits with lowest e-values, sequences most closely matched those of *Theobroma cacao* (10,050 hits), with *Vitis vinifera* having the second highest number of top hits (6490) (Fig. 2). The higher similarity to *T. cacao* sequences is a reflection of the phylogenetic relatedness of these species to *D. viscosa* [29].

There is currently a lack of genomic information available for *Dodonaea*, as demonstrated by the lack of hits to *Dodonaea* sequences in the BLAST results. The majority of BLAST hits were to agriculturally relevant species, such as *Vitis vinifera*, *Theobroma cacao*, and *Glycine max,* that have had a plethora of genomic data generated for them and so hits to these species are much more likely, as found in other plant transcriptome characterisation studies [10, 15, 30].

This study acts as the starting point of exploration into adaptation across environmental gradients in *Dodonaea viscosa* and, as such, we are interested in identifying genes that may be under differential selection among contrasting climatic regimes. In terms of seeking out genes under selection over a latitudinal gradient where rainfall and temperature clines are steep, genes related to water balance and response to drought are obvious candidates. One such group of genes is the aquaporins, which are involved in membrane permeability, are ubiquitous amongst living organisms, and have been the subject of a number of in-depth functional studies in plants [31, 32]. Within the transcriptome assembly presented here, 16 contigs had significant homologies with aquaporin or probable aquaporin genes (Table 1), potential targets for future genetic adaptation studies. Abscisic acid (ABA) has also been shown to be involved in water stress responses [33] and ABA signal transduction is interconnected with aquaporin function [34]. Genes involved in ABA production and function are therefore also good candidate genes for investigating adaptation to water-related stress. 29 such genes had homologous sequences within our assembled transcriptome, five of which had “response to water deprivation” gene ontologies assigned (Additional file 1).

**Table 1:** Contigs with significant Blast hits to aquaporin genes

Contig number	Description	Minimum e-Value	Gene ontologies
11,322	Aquaporin nip1-2	5.78e^−103^	P^1^:arsenite transport; P:response to hypoxia; C^2^:integral to membrane; F^3^:arsenite transmembrane transporter activity; F:lactate transmembrane transporter activity; P:hydrogen peroxide transmembrane transport; P:lactate transmembrane transport; P:response to arsenic-containing substance; C:endoplasmic reticulum; C:plasma membrane
17,499	Probable aquaporin sip2-1-like	1.17e^−128^	C:integral to membrane; C:endoplasmic reticulum; P:transport; C:plasma membrane; F:transporter activity; P:response to arsenic-containing substance
21,612	Aquaporin nip2-1-like	7.68e^−45^	P:silicate transport; C:Casparian strip; C:integral to membrane; F:silicate transmembrane transporter activity
30,905	Aquaporin nip2-1-like	9.79e^−109^	C:Casparian strip; C:integral to membrane; F:silicate transmembrane transporter activity; P:silicate transport
37,229	Aquaporin nip6-1	3.60e^−28^	F:borate transmembrane transporter activity; F:urea transmembrane transporter activity; F:glycerol transmembrane transporter activity; P:myo-inositol hexakisphosphate biosynthetic process; C:integral to membrane; P:borate transmembrane transport; F:water channel activity; P:urea transmembrane transport; P:glycerol transport; P:cellular response to boron-containing substance levels; C:plasma membrane
50,450	Aquaporin nip2-1-like	1.03e^−28^	C:Casparian strip; C:integral to membrane; F:silicate transmembrane transporter activity; P:silicate transport
6446	Aquaporin tip4-1-like	5.03e^−131^	P:water transport; C:plant-type vacuole membrane; C:central vacuole; P:transmembrane transport; C:integral to membrane; F:water channel activity; P:cytokinin mediated signaling pathway
59,060	Aquaporin	7.44e^−45^	P:transport; C:integral to membrane; F:transporter activity
60,372	Aquaporin pip	3.12e^−32^	P:transport; C:integral to membrane; F:transporter activity
64,509	Probable aquaporin nip7-1-like	2.16e^−20^	P:borate transmembrane transport; F:borate transmembrane transporter activity; P:purine nucleobase transport; C:integral to membrane; F:water channel activity
68,886	Aquaporin pip1 1	1.11e^−47^	P:response to water deprivation; C:chloroplast envelope; C:vacuole; C:anchored to plasma membrane; P:water transport; C:integral to membrane; P:response to salt stress; C:mitochondrion; F:transporter activity
72,268	Probable aquaporin pip1-2-like	1.34e^−35^	P:brassinosteroid biosynthetic process; P:response to water deprivation; C:mitochondrion; P:response to salt stress; C:integral to membrane; P:acetyl-CoA metabolic process; F:water channel activity; C:chloroplast envelope; P:cellular response to iron ion starvation; P:iron ion transport; F:protein binding; P:water transport; C:anchored to plasma membrane; C:vacuole; P:sterol biosynthetic process
74,405	Aquaporin pip1 1	3.98e^−38^	P:transport; C:integral to membrane; F:transporter activity
90,132	Aquaporin nip1-2-like	8.07e^−28^	C:membrane
97,487	Probable aquaporin nip-type-like	3.39e^−60^	C:membrane; P:transport; F:transporter activity
1354	Aquaporin	2.55e^−134^	P:response to water deprivation; C:chloroplast envelope; C:vacuole; C:anchored to plasma membrane; P:water transport; C:integral to membrane; P:response to salt stress; C:mitochondrion; F:transporter activity

^1^ *P* biological process, ^2^ *C* cellular component, ^3^ *F* molecular function

**Fig. 3:**
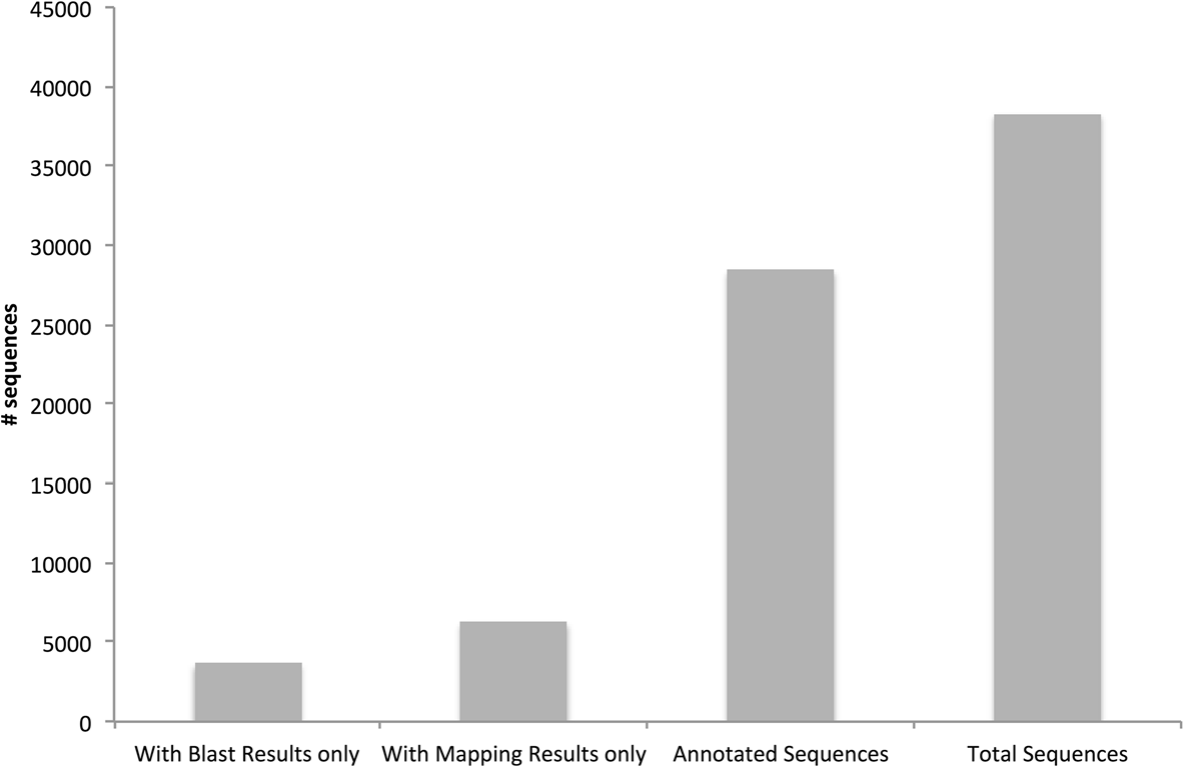
Gene Ontology (GO) annotation outcome. Blast2GO was used to attempt to annotate the 38,311 contigs that had significant BLAST hits (≤1e^−5^) to plant species. The number of contigs that had no GOs assigned (with Blast results only), mapping results only (GO terms retrieved but reliable functions not selected), and that were fully annotated are shown

**Fig. 4:**
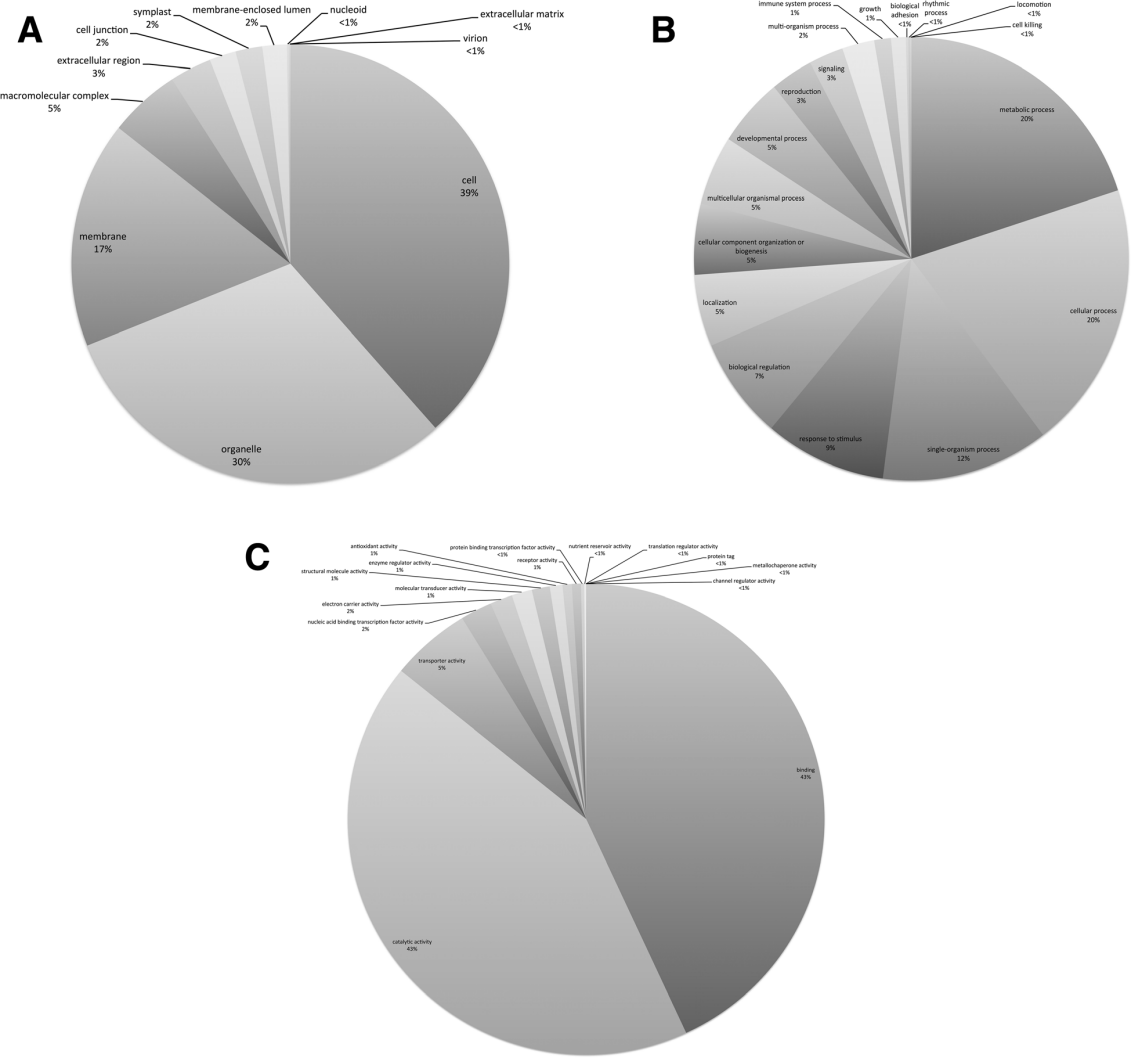
Distribution of assembled *Dodonaea viscosa* ssp. *angustissima* and ssp. *spatulata* contigs across three main GO categories. Proportions of contigs assigned various GO terms under the cellular component (CC) category (**a**), the biological process (BP) category (**b**), and the molecular function (MF) category (**c**). The number of contigs with annotations in the CC, BP, and MF categories were 33,789, 23,615 and 28,040 respectively

Gene ontology (GO) annotation was performed on the 38,311 contigs using the CLC Blast2GOpro plugin. Of these contigs, 28,440 (74 %) were annotated, with a total of 85,444 GO terms assigned (Fig. 3). The number of GOs assigned per sequence ranged from 1 to 66. GO terms fit under three broad categories: biological processes (BP), cellular components (CC), and molecular function (MF). The number of assignments per category was as follows: Cellular components: 33,789 GOs; Biological processes: 23,615 GOs; Molecular function: 28,040 GOs (Fig. 4). It is interesting to note that, under the biological processes category, 8236 contigs were assigned GO terms relating to ‘response to stimulus’ (Fig. 4b). Of these, a number of GO terms assigned relate to a response to an environmental stressor. For example, response to salt stress (assigned 689 times), response to cold (417), defence response (414), response to water deprivation (308), response to oxidative stress (291), and response to high light intensity (255) were all abundantly assigned. Polymorphisms in and/or differential expression of these genes between populations along an environmental gradient could be an indication of adaptation to local conditions and so should inform future studies into such adaptation.

Nine hundred two enzyme codes were assigned to 5070 contig sequences, which were included in 146 different KEGG pathways (Additional file 2). The most highly represented pathways were “Purine metabolism”, “Starch and sucrose metabolism” and “Phenylalanine metabolism” with 561, 548, and 316 assigned contigs respectively (Table 2). This mirrors the findings of a recent annotation of the Aleppo pine (*Pinus halepensis* Mill.) transcriptome, where 419 of their assembled contigs were assigned to the “Purine metabolism” pathway and 399 contigs were assigned to the “Starch and sucrose metabolism” pathway [26]. Given that our transcripts were from leaf tissue, it is not surprising to find that so many are involved in the metabolism of compounds such as amino acids, starch, and sugars. “Phenylpropanoid biosynthesis” was also a well-represented pathway, with 290 sequences assigned to it. Phenylpropanoids, a diverse family of secondary metabolites synthesised from phenylalanine, play vital roles in a wide range of responses to environmental stimuli in plants, such as UV photoprotection, attraction of insect pollinators, and defence against infection and herbivory, as well as being involved in reproduction and the internal regulation of cell physiology and signalling [35]. Again, as leaf tissue requires protection from UV light and is the main site of herbivory, a high prevalence of gene transcripts involved in phenylpropanoid synthesis is as expected.

**Table 2:** Top 10 KEGG pathways represented by contig sequences

Pathway	Number of sequences
Purine metabolism	561
Starch and sucrose metabolism	548
Phenylalanine metabolism	316
Phenylpropanoid biosynthesis	290
T cell receptor signalling pathway	262
Pyrimidine metabolism	212
Glycolysis/Gluconeogenesis	210
Flavonoid biosynthesis	196
Amino sugar and nucleotide sugar metabolism	189
Glycerolipid metabolism	187

Of the 38,311 contig sequences, 28,165 had InterPro protein annotations. The most commonly occurring protein region was a pentatricopeptide repeat (PPR), with 13,692 occurrences (Table 3). PPR is a 35-amino-acid motif, first identified in *Arabidopsis thaliana*, that occurs in tandem arrays [36]. Proteins containing PPRs are particularly prevalent in the plant kingdom and they are mainly involved in organelle gene expression through RNA binding, editing [37], splicing, and stability (reviewed in [38]). Such a high prevalence of transcripts coding for PPRs in the leaf transcriptome of *D. viscosa* is therefore unsurprising, as a high concentration of chloroplasts would determine the presence of such proteins. Other commonly occurring domains and sites identified in our contigs include the highly conserved protein kinase domain (6014 occurrences), which contains the catalytic function of protein kinases; proteins belonging to the cytochrome P450 family (5079 occurrences), a diverse group of enzymes involved in the oxidation of organic substances; and leucine-rich repeat regions (2952 occurrences), a repeating stretch of 20–29 amino acids that form an α/β horseshoe fold [39] and are involved in the formation of protein-protein interactions [40] (Table 3).

**Table 3:** Top 20 InterPro scan annotations

IPR accession	Description	Type	Occurrence
IPR002885	Pentatricopeptide repeat	Repeat	13,692
IPR000719	Protein kinase domain	Domain	6014
IPR001128	Cytochrome P450	Family	5079
IPR001611	Leucine-rich repeat	Repeat	2952
IPR027417	P-loop containing nucleoside triphosphate hydrolase	Domain	2150
IPR000477	Reverse transcriptase domain	Domain	2084
IPR011009	Protein kinase-like domain	Domain	2058
IPR002290	Serine/threonine- /dual specificity protein kinase, catalytic domain	Domain	1744
IPR012337	Ribonuclease H-like domain	Domain	1652
IPR001680	WD40 repeat	Repeat	1504
IPR008271	Serine/threonine-protein kinase, active site	Active site	1491
IPR001841	Zinc finger, RING-type	Domain	1314
IPR001878	Zinc finger, CCHC-type	Domain	1233
IPR001650	Helicase, C-terminal	Domain	1153
IPR011990	Tetratricopeptide-like helical	Domain	1104
IPR001752	Kinesin, motor domain	Domain	1040
IPR020683	Ankyrin repeat-containing domain	Domain	1036
IPR001245	Serine-threonine/tyrosine-protein kinase catalytic domain	Domain	915
IPR003657	DNA-binding WRKY	Domain	904
IPR001584	Integrase, catalytic core	Domain	894

In this study, only leaf tissue was collected for RNA extraction. This was for two reasons: 1) leaves in this species demonstrate a morphological cline [5, 6] and so are a tissue of interest when looking to answer questions about adaptation, and 2) it simplified the collection and extraction process. Gene activity varies between tissue type as well as varying with the time of day and across seasons. As each tissue type will have a different set of active genes, the transcriptome we have characterised is specific to the leaf tissue in this species. To gain a more holistic view and to include genes that are not transcribed in leaf tissue, other tissue types would also need to be sampled. Despite this, a broad variety of genes with diverse functions is represented in our contigs, as demonstrated by the BLAST results, gene ontology annotation, and KEGG analysis.

### Subspecies comparison

**Fig. 5:**
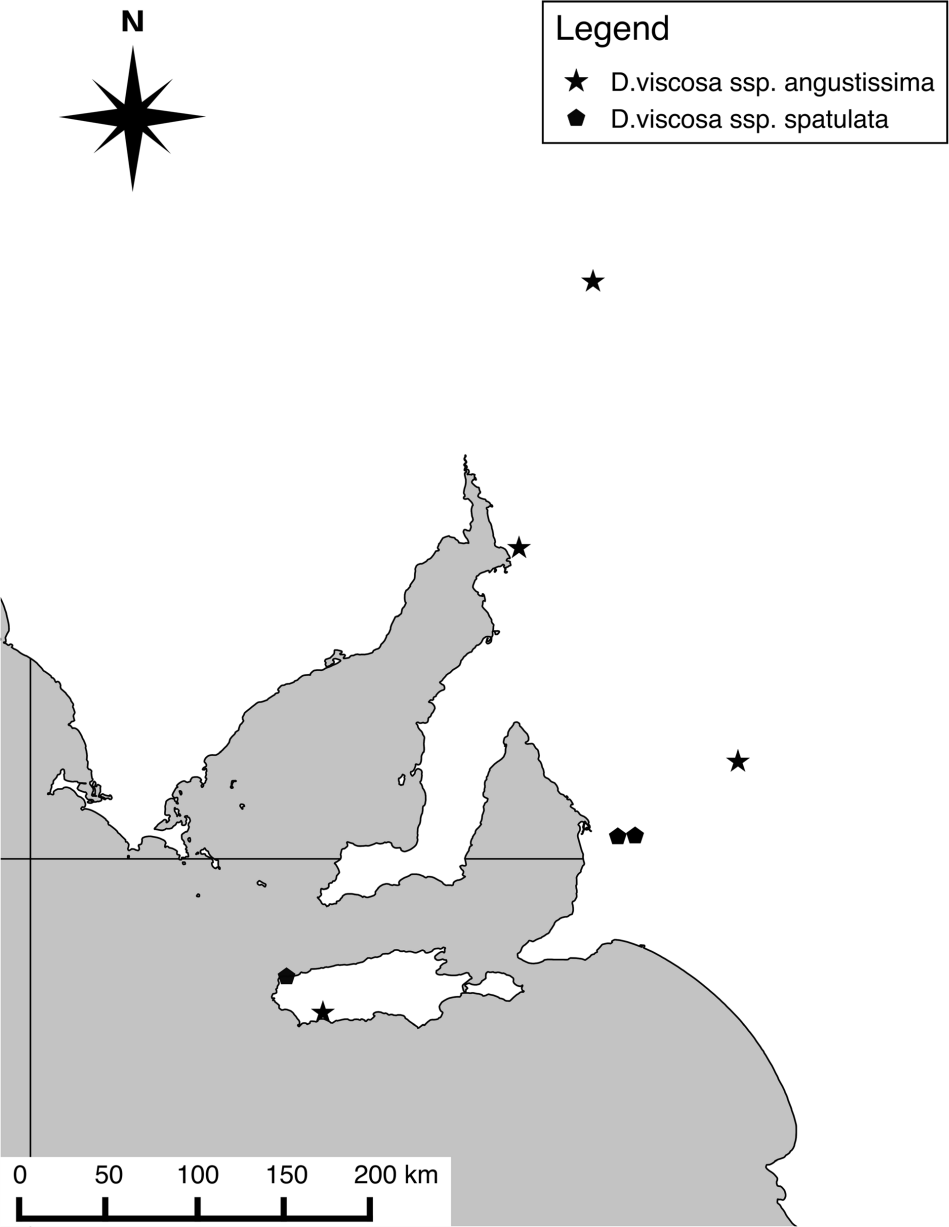
Map of *Dodonaea viscosa* sample population locations. The Adelaide-geosyncline region with the Southern Ocean to the south. Locations of the four *D.viscosa* ssp. angustissima and 3 ssp. spatulata populations sampled for RNA extraction are indicated by star and pentagon symbols respectively

The two subspecies within our sample, *D. viscosa* ssp. *angustissma* and ssp. *spatulata*, are distinguishable morphologically mainly by leaf shape [4]. However, this distinction appears not to be absolute and intergrades between forms can be found [4 and personal observation]. Their ranges within the study region overlap slightly, with ssp. *angustissima* restricted mostly to the hotter, drier north and ssp. *spatulata* in the cooler, wetter south, with the exception of sympatric populations on Kangaroo Island (Fig. 5). The degree of genetic differentiation between the subspecies is unknown. Here, we sought to find fixed genomic differences between the transcriptomes of the two subspecies. Mapping the ssp. *spatulata* reads onto a set of consensus ssp. *angustissima* sequences and then looking for polymorphisms between the reference sequences and the mapped reads resulted in the identification of 8494 fixed SNP differences over 3979 shared contigs. The transition/transversion ratio of these SNPs was 1.34, showing a transition bias. This is as expected in the sense that transitions occur more readily due to the molecular mechanisms underlying them and is comparable to the transition/transversion ratio of 1.65 found by [10] in their comparison of big sagebrush (*Artemisia tridentata*) subspecies transcriptomes.

A wide diversity of GO terms were assigned to the subset of shared contigs across the broad categories of cellular components, biological processes and molecular functions (Additional file 3), suggesting that diversification of the subspecies has occurred over a wide range of genes and for a number of traits. Further investigation into fixed differences by, for example, large scale screening for these genes within populations of the two subspecies (as well as other *D.viscosa* subspecies) using targeted gene sequencing technologies such as hybrid capture may provide an insight into the genes involved in adaptation and possibly the mechanisms involved in reproductive isolation leading to speciation.

### Variant detection and marker development

All cleaned reads from both subspecies were mapped onto the set of 38,311 contigs with significant blast hits to Viridiplantae gene sequences. Of the 227,376,588 reads, 165,172,256 (72.64 %) mapped to the reference sequences. 248,235 SNPs were identified across 25,270 contigs, with a transition/transversion ratio of 1.38. Stringent quality score settings ensured a high average SNP quality score of 44.65 and average coverage per SNP was 362.04.

In the 19,210 contigs that were input into ESTScan, 17,388 coding regions were identified across 16,982 contigs (a number of longer contigs were identified as containing multiple coding regions). After mapping the reads from all populations onto these contigs, 140,246 SNPs were identified across 9400 contigs. Of these, 56,903 SNPs distributed over 2343 contigs were identified as non-synonymous. Non-synonymous SNPs result in amino acid changes in the translated polypeptide and, as such, can be targets of selection. Therefore, this identified set of 2343 contigs may be of particular interest in future studies when looking for signatures of selection.

SNPs are yet to be validated as markers suitable for investigating *D. viscosa* ecology and adaptation and future work is planned to this end. In this study, only two individuals per population were sampled making it difficult to assess allelic variation between populations. However, previous studies have relied on fewer individuals than this when identifying variants from transcriptome data [14, 16]. A more extensive population screening of these potential markers is required, with several individuals per population, in order to measure allele frequencies and fixed differences between populations. A population study using targeted hybrid-capture of the polymorphic loci identified here is in progress in an attempt to uncover signals of selection along an environmental gradient as well as demographic history in this species. Beyond this, the resource could be utilised in linkage mapping and gene-based association studies within *D. viscosa*, as well as for comparative genomics. This transcriptome could also serve as a relevant genetic resource more widely for the currently under-represented *Dodonaea* genus.

## Conclusions

Using a single lane of an Illumina HiSeq^TM^ 2000 sequencing run we have generated an extensive genomic resource dataset, providing a broad characterisation of expressed genes in the leaves of *D. viscosa*, as well as identifying a large set of genetic SNP markers for future population genetic analysis of this species. Our results, along with several other recent studies, indicate that short reads from Illumina sequencing can be effectively assembled to provide a characterisation of the transcribed genes within a non-model species [8, 9, 11, 13, 15, 17, 25, 30]. The diversity of genes identified through BLAST searches, gene ontological annotations, KEGG pathways, and protein domain screening demonstrates that we have successfully assembled a robust set of transcriptome sequences representing a wide range of genes of functional significance that are expressed within the leaf tissue of *D. viscosa.* This study provides an extensive genetic resource that can be utilised to delve deep into the ecology and evolution of a species/genus currently lacking in such resources.

## Methods

### Source populations and leaf material collection

Leaves were field collected from seven populations in South Australia; Three *D. viscosa* ssp. *spatulata* populations and four *D. viscosa* ssp*. angustissima* populations. Sampling sites traversed a latitudinal gradient in South Australia (Fig. 5). Along this gradient average annual rainfall and mean maximum temperature ranges from >700 mm and 19 °C in the south to <200 mm and 25 °C in the north, respectively. Populations varied in size at each site from fewer than 10 individuals to more than 50 individuals. For each population, young leaves were harvested from two individuals, packed into falcon tubes and stored in liquid N_2_ until extraction. Sampling was restricted by accessibility as we had to be able to drive as close to the collection sites as possible in order to minimise time between picking the leaves and getting them into the liquid N_2_ in order to keep RNA degradation to a minimum. We also selectively picked young, actively growing leaves to increase RNA yield.

### RNA extraction

RNA extraction was performed on 2 g of frozen leaf tissue per population sample using a modified CTAB extraction method [41]. The extracted RNA was analysed for yield and quality using Agilent 2200 TapeStation (Agilent Technologies, Santa Clara, CA) prior to sending to the Australian Genome Research Facility (AGRF) for cDNA synthesis and sequencing.

### cDNA library preparation and Illumina sequencing

Library preparation and sequencing were performed by AGRF, Melbourne, Australia. Illumina’s TruSeq RNA sample preparation protocol was followed for the preparation of mRNA-seq libraries. Briefly, polyadenylated mRNA was isolated from total RNA using oligo dT magnetic beads. mRNA is then fragmented and synthesised using SuperScript II Reverse Transcriptase (Invitrogen). 3′ adenylation of the resulting cDNA fragments then allows for ligation of the sequencing adapters. The resultant libraries were amplified via 12 cycles of PCR. Libraries were assessed using Agilent’s Bioanalyser DNA 1000 chip and qPCR was used to quantify the libraries prior to normalising to 2 nM and pooling. Cluster generation for paired end sequencing was performed on an Illumina cBot following the manufacturer’s protocol. Paired end sequencing was performed on one lane of an Illimina HiSeq with 208 cycles (101, 6 and 101 cycles for read 1, index read, and read 2 respectively) according to the manufacturer’s protocol. Post run processing, including demultiplexing and generation of Fastq files, was performed using Illumina’s CASAVA pipeline 1.8.2.

### Quality control

Following sequencing, quality control of the sequencing reads was performed in CLC. Duplicate reads were removed using the ‘remove duplicates’ plugin followed by trimming using the ‘trim sequences’ tool with setting as follows: removal of low quality sequences: limit = 0.05; removal of ambiguous nucleotides: maximum 2 nucleotides allowed; removal of sequences on length: minimum length 80 nucleotides.

### De novo assembly

Following the QC steps, *de novo* assembly of high quality reads was carried out using the *de novo* assembly algorithm within the CLC genomics workbench. This algorithm utilises *de Bruijn* graphs to represent overlapping reads. The following settings were used: automatic bubble size (50), minimum contig length of 201, automatic word size (23), perform scaffolding, and auto-detect paired distances. The mapping mode was also used, whereby all reads were mapped back to the assembled contigs and contigs were then updated as a result of the mapping. The mapping settings were as follows: mismatch cost 2, insertion cost 3, deletion cost 3, length fraction 0.9, similarity fraction 0.8. Following *de novo* assembly any duplicate sequences were removed using the ‘remove duplicates’ CLC plugin.

To validate the CLC assembly, reads were also passed through the Trinity *de novo* assembly pipeline using default parameters [28]. The output contigs were then imported into CLC where duplicates were removed using the ‘remove duplicates’ plugin. As Trinity generates putative splice variants only the longest contig per component (equivalent to gene) was selected using a custom script. Reads were then mapped back to these contigs in CLC using the same settings as the mapping to CLC contigs. Reciprocal mappings between the two sets of contigs were then performed in CLC in order to compare the two assemblies.

### Sequence annotation

#### BLAST

Assembled contigs were compared to the public NCBI NR protein database using BLASTx in order to identify putative protein homologies. Default parameters were used. An *e*-value cut-off of ≤1e^−5^ was used in order to restrict results to the most significant matches. As our samples were field collected there was a high chance of the presence of non-plant RNA from e.g. parasites and endophytes. Therefore, MEGAN 4 (http://ab.inf.uni-tuebingen.de/software/megan4/) (Huson et al. [42]) was used to create a list of contigs that were assigned to non-plant species. These could then be excluded from further analyses.

#### BLAST2GO

The BLASTx output was imported into CLC and gene ontology (GO) terms were assigned using the “mapping” and “annotation” tools in the Blast2GO plugin.

#### KEGG

GO terms were directly mapped to their enzyme code equivalents in the BLAST2GO Java application (http://www.blast2go.com/b2ghome) in order to generate enzyme code and KEGG (Kyoto Encyclopedia of Genes and Genomes) pathway annotations thus identifying which metabolic pathways the gene products are involved in.

#### InterPro scan

Functional analysis of the translated protein sequences was carried out using InterProScan via the BLAST2GO Java application. InterPro provides functional analysis of proteins by classifying them into families and predicting domains and important sites (http://www.ebi.ac.uk/interpro/).

### Comparison of subspecies

In order to look for fixed differences between the sampled subspecies, a number of steps were taken. The reads from ssp. *angustissima* populations only were mapped against the set of contigs with significant BLAST hits to land plant species in CLC with the following settings: mismatch cost: 2, insertion cost: 3, deletion cost: 3, length fraction: 0.7, similarity fraction: 0.8**.** Consensus sequences were then extracted from this mapping, using ambiguity codes where alternate alleles were present. These consensus sequences were then used as the reference for the ssp. *spatulata* reads to be mapped to with the same settings as above. This enabled us to specifically call variants between the ssp. *angustissima* consensus sequences and the mapped ssp. *spatulata* reads making the identification of fixed differences between the subspecies more straightforward. Quality-based variant detection in CLC was then run on this mapping in order to identify variants between the two subspecies with the following settings: neighbourhood radius: 10, maximum gap and mismatch count: 5, minimum neighbourhood quality: 20, minimum central quality: 30, ignore non-specific matches, ignore broken pairs.

### Single Nucleotide Polymorphism (SNP) discovery

In order to discover SNPs between our samples, all reads that made it through the QC step were mapped on to the contig sequences with significant BLAST hits to members of the *Viridiplantae* clade using the ‘map to reference’ tool in CLC with the following settings: mismatch cost 2, insertion cost 3, deletion cost 3, length fraction 0.7, similarity fraction 0.8, global alignment, auto-detect paired distances, ignore non-specific matches. The quality-based variant detection tool in CLC was then employed to identify variants using the following settings: neighbourhood radius: 10, maximum gap and mismatch count: 5, minimum neighbourhood quality: 20, minimum central quality: 30, ignore non-specific matches, ignore broken pairs, minimum coverage: 20, minimum variant frequency: 20 %, maximum expected alleles: 2.

In order to assess whether identified SNPs were synonymous or non-synonymous, ESTScan (http://estscan.sourceforge.net) was used to identify coding regions within the assembled contigs. This program employs a hidden Markov model in order to detect and extract coding regions from sequence data [43]. A subset of 19,210 contigs identified as having significant BLAST hits (≤1e^−5^) to land plant species using MEGAN with a low complexity threshold of 0.44 (to ensure low complexity sequences were excluded) was used in this analysis. ESTScan relies upon score matrices specific to the study species. We used the *Arabidopsis thaliana* score matrix provided with the software [10]. Annotations indicating the predicted coding sequence were then added to the contigs using the ESTScan output. Raw reads from all samples were then mapped on to these annotated contigs in CLC with the same settings as in previous mappings. Quality-based variant detection was then run, again with the same settings as in previous variant detection. Resultant SNPs were identified as either synonymous or non-synonymous in the CLC output.

### Ethics statement

No ethics approval was required for this study. All relevant permits and approvals were obtained for the field collections carried out in this study. Collection sites were within national parks and conservation parks managed by the South Australian Department of Environment, Water and Natural Resources. No protected species were sampled.

### Availability of supporting data

The raw sequencing data sets supporting the results of this article are available in the NCBI SRA repository [accession numbers: SRR1914329, SRR1914332, SRR1914333, SRR1914334, SRR1914335, SRR1914337, SRR1914338, http://www.ncbi.nlm.nih.gov/bioproject/?term=dodonaea%20viscosa].

The assembled transcriptome contigs have been deposited at www.labarchives.com, DOI: 10.6070/H4NS0RW1.

## Additional files

Additional file 1:
**Lists of contigs with significant BLAST hits (e-value < 1e-5) to abscisic acid andaquaporin related genes.** (XLSX 49 kb) Additional file 2:
**Output from KEGG analysis of contigs, listing identified pathways that gene products areinvolved in.** (XLSX 120 kb) Additional file 3:
**Gene Ontology terms assigned to the contigs with significant BLAST hits to plant genes,listed under the categories 'biological processes', 'cellular components', and 'molecular functions'.** (XLSX 13 kb)
